# High expression of MCM10 is predictive of poor outcomes in lung adenocarcinoma

**DOI:** 10.7717/peerj.10560

**Published:** 2021-02-03

**Authors:** Mingrui Shao, Shize Yang, Siyuan Dong

**Affiliations:** Department of Thoracic Surgery, The first hospital of China Medical University, Shenyang, Liaoning, China

**Keywords:** Lung adenocarcinoma, MCM10, Biomarker, Prognosis

## Abstract

**Backgrounds:**

Lung adenocarcinoma is a complex disease that results in over 1.8 million deaths a year. Recent advancements in treating and managing lung adenocarcinoma have led to modest decreases in associated mortality rates, owing in part to the multifactorial etiology of the disease. Novel prognostic biomarkers are needed to accurately stage the disease and act as the basis of adjuvant treatments.

**Material and Methods:**

The microarray datasets GSE75037, GSE31210 and GSE32863 were downloaded from the Gene Expression Omnibus (GEO) database to identify prognostic biomarkers for lung adenocarcinoma and therapy. The differentially expressed genes (DEGs) were identified by GEO2R. Functional and pathway enrichment analysis were performed by Kyoto Encyclopedia of Genes and Genomes and Gene Ontology (GO). Validation was performed based on 72 pairs of lung adenocarcinoma and adjacent normal lung tissues.

**Results:**

Results showed that the DEGs were mainly focused on cell cycle and DNA replication initiation. Forty-one hub genes were identified and further analyzed by CytoScape. Here, we provide evidence which suggests MCM10 is a potential target with prognostic, diagnostic and therapeutic value. We base this on an integrated approach of comprehensive bioinformatics analysis and in vitro validation using the A549 lung adenocarcinoma cell line. We show that MCM10 overexpression correlates with a poor prognosis, while silencing of this gene decreases aberrant growth by 2-fold. Finally, evaluation of 72 clinical biopsy samples suggests that overexpression of MCM10 in the lung adenocarcinoma highly correlates with larger tumor size. Together, this work suggests that MCM10 may be a clinically relevant gene with both predictive and therapeutic value in lung adenocarcinoma.

## Introduction

Lung Adenocarcinoma is one of the most prevalent and lethal cancers worldwide. The most recent global estimates suggest an annual incidence rate of 1.8 million individuals (Barta, Powell & Wisnivesky, 2019). In the US alone, there are nearly 700 diagnoses per day (De Groot et al., 2018), and unlike small cell lung cancers which occurs almost exclusively in smokers, lung adenocarcinoma affects smokers and non-smokers alike (Videtic et al., 2003). While remarkable progress has been made in developing new treatments and intervention strategies, the 5-year survival is still less than 15% (De Groot et al., 2018). Treatment is often dependent on the stage of diagnosis, which unfortunately rarely occurs at an early stage (Blandin Knight et al., 2017). Thus, identifying the stage and subtype of lung adenocarcinoma is vitally important for prognosis and management of the disease. The variability in survival rate, even within stages, has naturally led to an intense examination of molecular alterations in various stages of the disease (Nikliński et al., 2001).

The identification of key molecules with aberrant expression patterns has been a crucial advancement in treating lung adenocarcinoma. The most common molecular markers for lung adenocarcinoma are epidermal growth factor receptor (EGFR) and Kirsten ras (KRAS), both of which may occur in nearly 30% of all cases (Testa, Castelli & Pelosi, 2018). Interestingly, EGFR mutations often occur in non-smokers while KRAS is more highly associated with smokers (Govindan et al., 2012). EGFR mutations alone can occur in a variety of ways, with the most common being L858R mutation or exon 19 deletion (Politi et al., 2006). Currently, there is a repertoire of tyrosine kinase inhibitors (TKIs) for adjuvant/neoadjuvant treatment of lung adenocarcinoma (Grigoriu, Berghmans & Meert, 2015). Non-smokers with an exon 19 deletion are known to respond to first generation TKI’s (Köhler & Schuler, 2013). Conversely, while there is a promising KRAS inhibitor for smokers with KRAS mutation (Canon et al., 2019), no FDA approved treatments currently exist. This highlights the potential benefits and current limitations in targeted therapies and the overall need for identifying novel prognostic and diagnostic molecular markers associated with lung adenocarcinomas.

**Figure 1:**
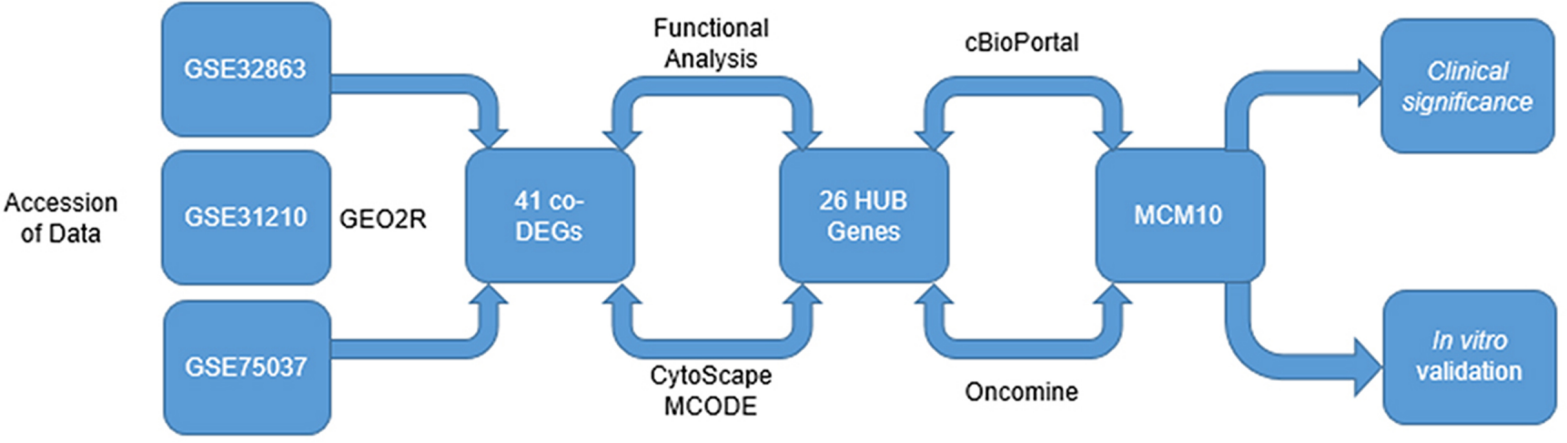
Integrated approach that lead to the identification of MCM10 in Lung Adenocarcinoma. Analysis started with three data sets take from GEO. A total of 41 CO-Degs and 26 HUB genes were identified from this data set. Ultimately, MCM10 was rationalized to be the most pertinent and evaluated further.

Here, we evaluate three unique sets of microarrays from the genome expression omnibus (GEO) to discover a novel molecular marker present in late stage adenocarcinomas (Fig. 1). We present evidence that Minichromosome Maintenance 10 (MCM10) is a prominent marker for late stage lung adenocarcinoma. MCM10 is a member of the MCM family of molecules. These proteins are highly conserved and essential for cellular division. In normal cells, MCM10 promotes DNA replication through interactions with other cell division cycle proteins, most notably CDC45 to initiate the pre-replication complex (Baxley & Bielinsky, 2017; Mughal, Mahadevappa & Kwok, 2019). Previous work has demonstrated other members of the MCM family, namely MCM2-7, are highly expressed in tumors (Liu et al., 2017; Nowińska & Dzięgiel, 2010). Furthermore, recent evidence has suggested that MCM10 is associated with late stage cervical and urothelial cancers (CITATION MAHADEVAPPA PMID: 30135378). We identify MCM10 as a crucial HUB gene over expressed in lung adenocarcinoma. Furthermore, we demonstrate over expression of MCM10 results in increases in proliferation rates and self-renewal capacities. Finally, we validate these results in clinical biopsy specimens and show that tumor size is proportional to MCM10 expression levels. It is our hope that these results provide a rationale for future drug development and ultimately may lead to viable treatment options.

## Materials and Methods

### Accession of data

Three unique expression microarrays were used for the present analysis: GSE32863 (Selamat et al., 2012), GSE31210 (Okayama et al., 2012) and GSE75037 (Girard et al., 2016). All datasets were downloaded from the Gene Expression Omnibus (GEO). GSE32863 and GSE75037 datasets were produced using Illumina HumanWG-6/Ref-8 v3.0 expression beadchip platform while GSE31210 used Affymetrix Human Genome U133 Plus 2.0 Array. The pertinent details of each dataset are presented in Table 1.

**Table 1:** Properties of data accessed from GEO.

GEO NO	Date	Cases (*n*=)		DEGS
		Stage I	Stage II–IV	
GSE75037	2016	50	33	305
GSE31210	2011	168	58	1853
GSE32863	2012	34	24	215

### Identifying DEG and HUB genes

Bioinformatic analysis was performed based on our previous protocols (Dong, Zhu & Zhang, 2020). Briefly, Differentially Expressed Genes (DEGs) were identified from the three accessed datasets using GEO2R with a cutoff criteria set to a *P*-value of <0.05 and log fold change >1. Values were averaged In the event probe sets did not contain exact gene symbols or two or more probe sets contained the same gene symbols. An interaction network between identified DEGS was constructed using The Search Tool for Retrieval of Interacting Genes (STRING) database (Szklarczyk et al., 2015). Cytoscape version 3.4.0 software was then used to visualize the output. Molecular Complex Detection (Bader & Hogue, 2003) (MCODE) was used to arrange the topology, cluster the connected genes, and search through the resulting network. Cutoffs for inclusion were MCODE scores >5, degree cutoff of 2, node score cutoff of 0.2, node density cutoff of 0.1, *k*-score of 2, and max depth of 100. HUB genes were initially selected if their degree was >10. Hub genes were initially selected if their degree of connectivity was >25. We then performed a functional analysis HUB genes using Cytoscape’s Biological Networks Gene Ontology (GO) (Maere, Heymans & Kuiper, 2005) (BiNGO) and validated these findings using the Database for Annotation, Integrated Discovery, and Visualization (Da Huang, Sherman & Lempicki, 2009) (DAVID).

### Kyoto Encyclopedia of Genes and Genomes and GO enrichment analyses of DEGs

The functional annotation tools version 6.7 of the DAVID (http://david.ncifcrf.gov) were used to extract biological information about our DEGs. GO was also used to annotate genes and further analyze their biological functions. The DAVID online database was used to analyze the function and biological process of the screened DEGs. *P* < 0.05 was considered to indicate statistical significance.

### Identification of MCM10 as a crucial HUB gene

Hub genes were further stratified by evaluating degree of mutation using cBioPortal’s Oncomine (Rhodes et al., 2004) plug in to search The Cancer Genome Atlas provisional sample set of 586 samples. Expression at various stages of cancer was evaluated using UCSC Xena cancer browser. MCM10’s relevance in cancer and in lung adenocarcinoma was further evaluated by again using Oncomine and UCSC XenaBrowser to identify transcript level mutations and expression levels. Finally, the correlation between MCM10 expression and overall survival was evaluated using the Kaplan–Meier plotter (Nagy et al., 2018).

### In vitro analysis of MCM10

Bioinformatics results were confirmed using in vitro experiments on A549 lung adeno carcinoma cells (Shanghai Cell Bank, Shanghai, China). The expression level of A549 was confirmed on Human Protein Atlas (http://www.proteinatlas.org/). A549 cells were cultured in RPMI 1640 medium supplemented with 1× penicillin/streptomycin (Gibco, Waltham, MA, USA) and 10% Fetal Bovine Serum (Gibco). RNA was extracted using TRIzol reagent (Invitrogen, Carlsbad, CA, USA) as per manufacturer’s recommendations. RNA was converted to cDNA using PrimeScript RT reagent kit (Takara Bio, Dalian, China) and reverse transcription was performed as per manufacturer’s recommendations. Transcription levels were evaluated using reverse transcription quantitative real time polymerase chain reaction (RT-qPCR) RR901A Premix Taq^™^ (TaKaRa Taq^™^ Version 2.0 plus dye). The MCM10 shRNA sequence was as follows: 3′-CCGGGACGGCGACGGTGAATCTTATCTCGAGATAAGATTCACCGTCGCCGTCTTTTT-5′ (Jikai, Shanghai, China). The PCR primers for MCM10 or GAPDH were as follows: MCM10 sense, 5′ GAAGAAGGTTACGCCACAGAG′ and reverse, 5′ TTTACAGGTTCCCAGGTCAAG3′; GAPDH sense, 5′-CAA TGACCCCTTCATTGACC-3′ and reverse, 5′- TGGAAGATGGTGATGGGATT-3′. PCR cycling conditions were 50 °C for 2 min, followed by 95 °C for 10 min and then 40 cycles for 95 °C for 15 s and 60 °C for 1 min. The 2^−ΔΔCt^ method was used to calculate the relative expression level by normalizing to GAPDH levels. Proliferation and colony formation assays were performed using the CCK8 cell counting kit (Dojindo Molecular Technologies, Rockville, MD, USA) on cultures initially seeded at 30,000 cells/mL. Results are displayed as mean (*n* = 3) ± SEM. Differences were considered statistically significant for *P* < 0.05.

### Validation in clinical samples

A total of 72 paired lung adenocarcinoma tissues and adjacent normal tissues were collected from patients with a median age of 60 undergoing surgery at our hospital. Collections took place between June 2018 and June 2019. MCM10 expression was detected using qPCR. The research was approved by the Ethics Committee of The First hospital of China Medical University (2018-88). Written informed consent was obtained from all of the patients.

## Results

### Identifying lung adenocarcinoma HUB genes

**Figure 2:**
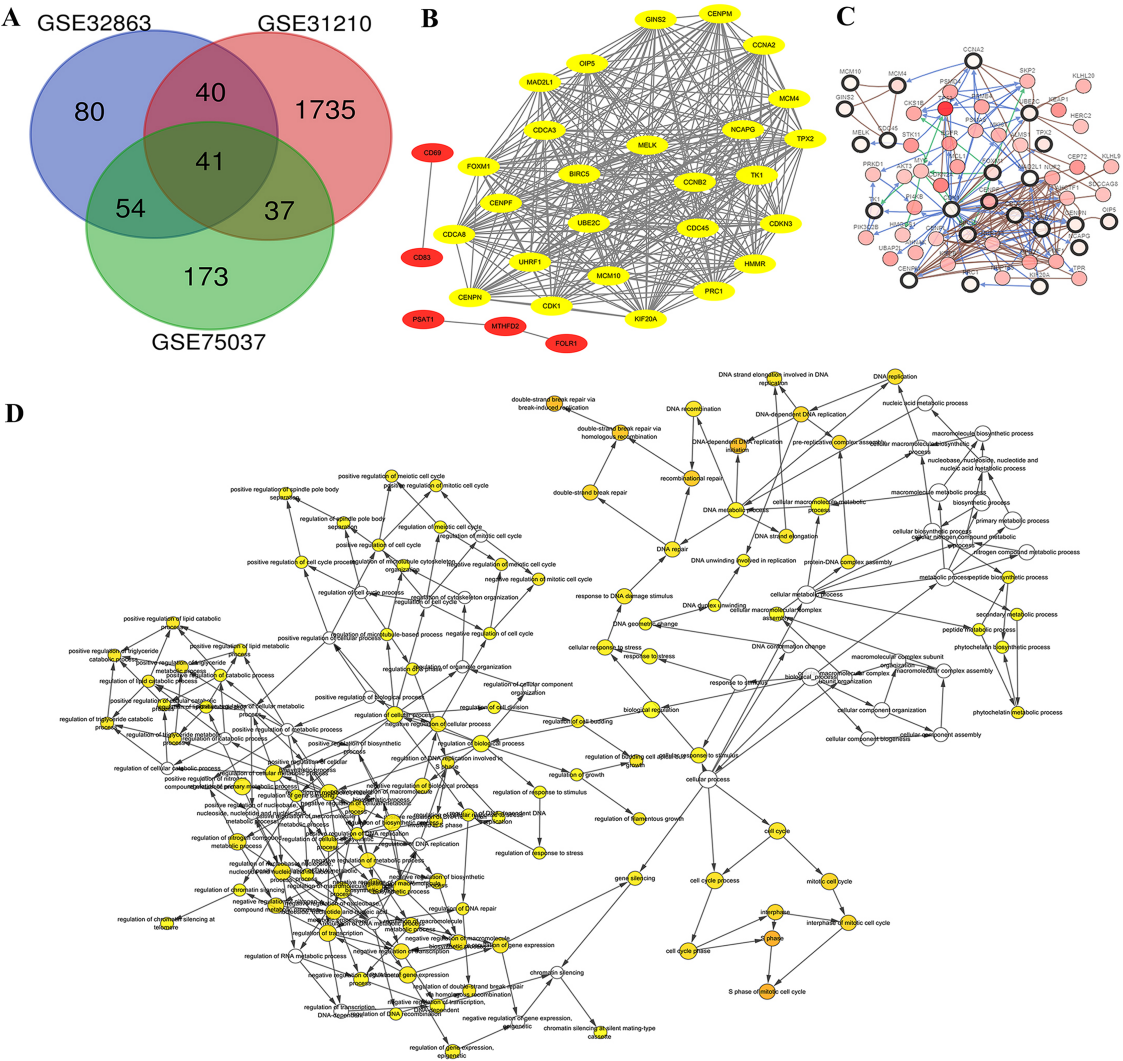
Identification of DEG and HUB genes in lung adenocarcinoma. (A) Three expression panels were probed: GSE32863, GSE31210 and GSE75037. A total of 41 CO-DEGs were found in the samples. (B) A total of 26 of these were deemed to be highly connected, with a cutoff of at least 25 connections. (C) The predicted pathways of the protein products were highly interactive. (D) Gene ontology analysis determined that there was an overrepresentation of proteins involved in S phase of mitosis and DNA replication.

Genome arrays provide invaluable data for bioinformatics-based analysis of DEGs. To fully identify and explore DEGs in lung adenocarcinoma samples we evaluated three separate arrays: GSE32863, GSE31210 and GSE75037. Characteristics of each are listed in Table 1. We chose these datasets because they were composed of matching samples of tumorous and non-tumorous tissues, allowing us to control for patient to patient differences. In all, we found 41 unique DEGs correlated with late stage lung adenocarcinomas in all three microarray samples (Fig. 2A). These 41 co-DEGs made the basis of our further analysis.

Gene IDs were entered into STRING database to predict protein-protein interactions and the resulting interactions were mapped using Cytoscape. We found ten of the genes had no connections, while five of the associated genes, CD69, CD83, PSAT1, MTHFD2 and FOLR1 were not highly connected (Fig. 2B, Red). The 26 additional genes showed high interconnectivity (Fig. 2B, yellow). These HUB genes have been found to be essential in cancer pathogenesis and progression. Further analysis via cBioPortal’s network application demonstrates high level of pathway interactivity. Interestingly, while many of the genes are highly connected, four of the HUB genes upregulated during lung adenocarcinoma (MCM10, MCM4, GINS2 and CD45) are predicted to interact nearly independently through direct binding mechanisms. We utilized the BiNGO application within Cytoscape to explore the HUB GO (Fig. 2D). While the HUB genes were highly connected in ontology, the most over represented were S phase of mitotic cell cycle, DNA replication initiation, and double strand break repair, insinuating a disruption in the cell cycle mechanism.

### MCM10 is a key HUB gene in lung adenocarcinoma

**Figure 3:**
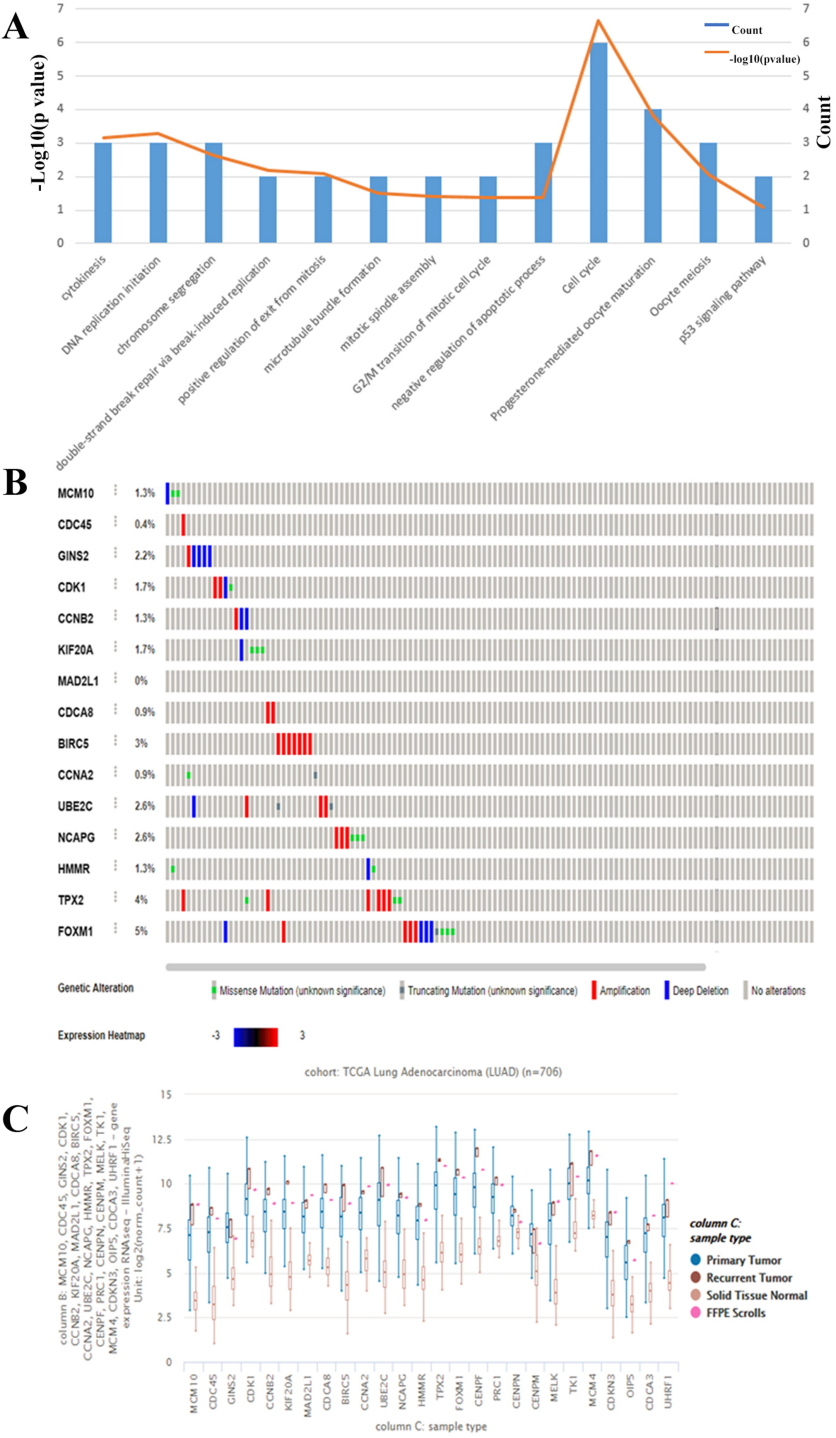
Evaluation of HUB genes in Lung Adenocarcinoma. (A) Of the 26 HUB genes, the majority are predicted to function in either cell cycle or progesterone-mediated oocyte maturation. The observation of cell cycle control corroborates the BiNGO analysis. (B) The highest 15 connected HUB genes were evaluated using Oncomine. All except for MAD2L1 have a known mutation in lung adenocarcinoma samples. (C) In addition, all of the hub genes are more highly expressed in primary and secondary tumors than in normal tissue.

We chose to further evaluate the function of the HUB genes which are over expressed in Lung Adenocarcinoma. Using DAVID, we found that the genes that are mostly over expressed are involved in cell cycle regulation, progesterone-mediate oocyte maturation, and negative regulation of apoptotic processes (Fig. 3A). To narrow down the list of potential key genes, we chose the top 15 most connected HUB genes. Of these, all except for MAD2L1 have a precedence of mutation in the Lung Adenocarcinoma TCGA Pan Cancer atlas (Fig. 3B). Furthermore, RNAseq analysis shows that each of these 15 are more highly expressed in both primary and recurrent tumors than in solid tumors (Fig. 3C). Based on the Cytoscape BiNGO analysis, it was evident that pertinent HUB genes should be connected to S phase of the mitotic cell cycle and DNA replication mechanisms (Fig. 2D). Based on an analysis of PathwayCommons, it was determined only four of the most connected HUB genes were involved in both pathways. Perhaps not surprisingly, many of these markers have been reported and extensively studied for their role in lung adenocarcinoma. However, of these four markers, MCM10 was found to be mutated the most (1.3% of samples), and therefore it was chosen as the most prominent target for the rest of the analysis. Through this, we have demonstrated that MCM10 may be a viable marker for lung adenocarcinoma.

### MCM10 expression is upregulated in lung adenocarcinoma

**Figure 4:**
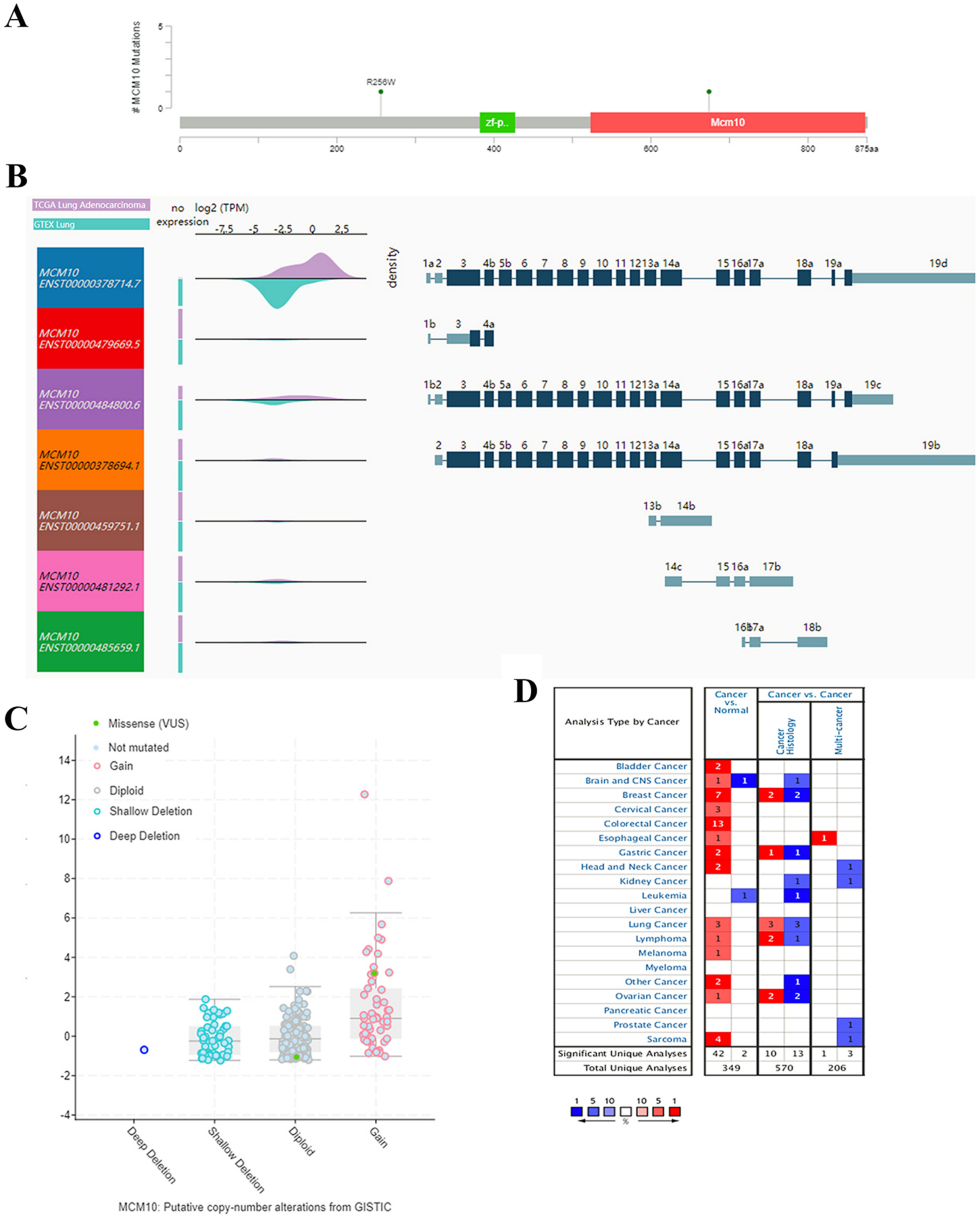
MCM10 is a clinically relevant biomarker in Lung Adenocarcinoma. (A) MCM10 levels are elevated in a variety of cancers, including lung cancers. (B and C) Analysis of MCM10 shows only a few mutations, but a high level of amplification, which may be due to overexpression of NMYC. The transcript MCM10-202 is the most highly expressed MCM10 transcript in lung adenocarcinoma tumor samples. (D) Expression profiles of MCM10 in 20 malignant tumor types are represented using the Oncomine database.

The results thus far have led to the hypothesis that MCM10 is a novel marker for lung adenocarcinoma. To further validate this theory, we further explored MCM10’s clinical value. This was accomplished by screening MCM10’s role in several organotypic cancers. We found that MCM10 is highly expressed in a variety of cancers in addition to lung cancer, including colorectal cancer and breast cancer (Fig. 4A). The incidences of cancer typically resulted in single copy alterations (i.e., gain). Interestingly, there were only a few mutations within the data set, suggesting that while MCM10 may be correlated with cancer pathogenesis, it may not be the leading cause (Fig. 4B). However, the mutations that do occur in MCM10 during lung adenocarcinoma arise from a missense mutation. We further evaluated our hypothesis by evaluating MCM10 transcript expression using the UCSC Xena Browser (Figs. 4C and 4D). Nearly all expressed transcripts are upregulated in lung adenocarcinoma samples, with the most prominently expressed transcript (MCM10-202) demonstrating a 2.5-fold increase in expression.

**Figure 5:**
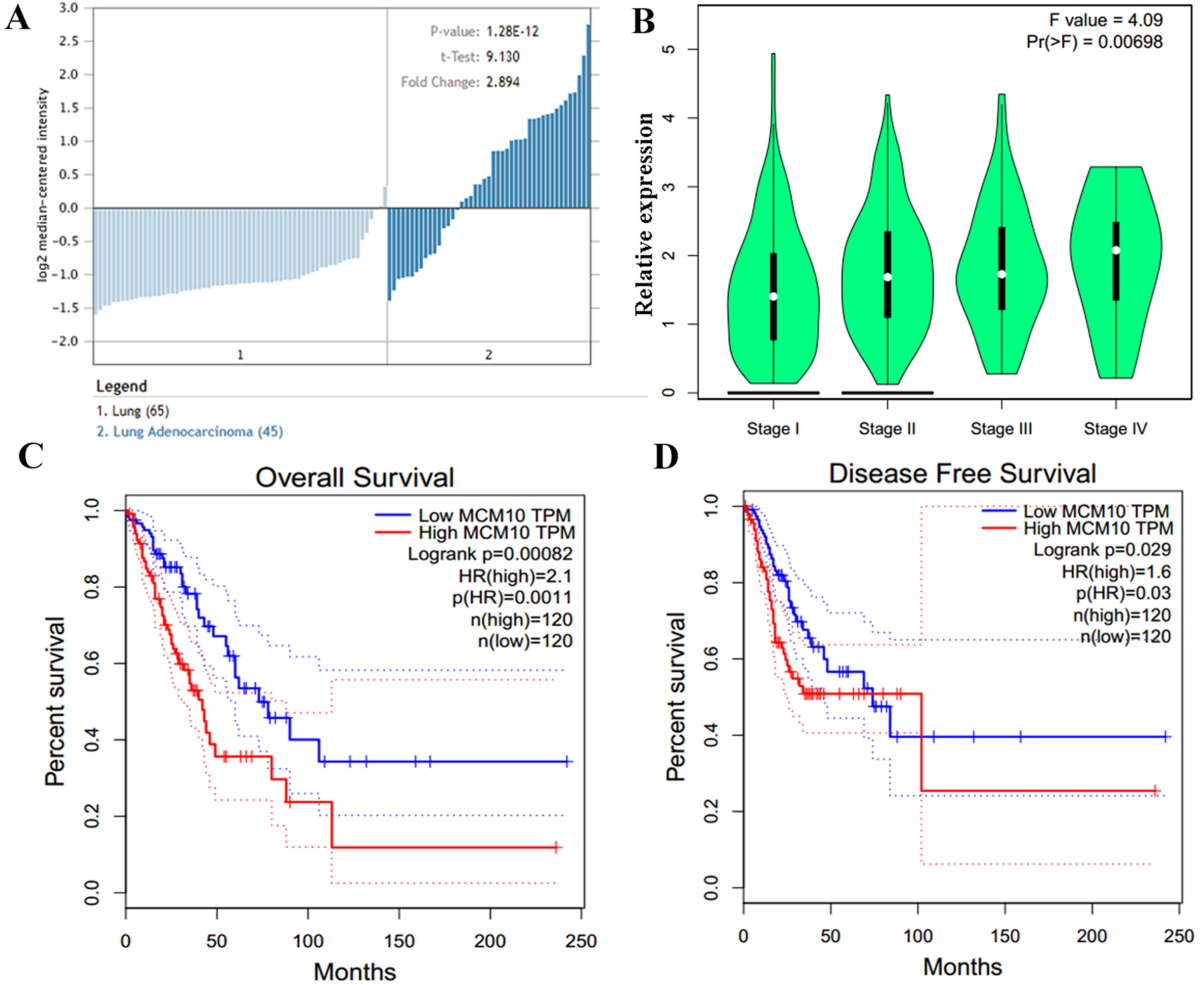
MCM10 expression is correlated with poor prognosis in Lung Adenocarcinoma. (A) MCM10 expression is, on average, nearly 3-fold higher in lung adenocarcinoma samples than in normal tissue. (B) The overall levels of MCM10 do not differ greatly between stages 1–4, however, the difference is statistically significant. (C and D) Both the 5-year survival and disease free survival of individuals with high MCM10 is much bleaker when the tumor presents with high MCM10 levels. The 5-year survival rates are 2.1-fold lower with high MCM10 expressions while the disease free survival is 1.6-fold higher.

The results thus far support the theory that MCM10 expression is a novel marker for lung adenocarcinoma. We corroborate this using Oncomine, which shows that there is an average of 2.9-fold increase in MCM10 in cancer samples compared to normal tissues (Fig. 5A). The normalized level of transcript expression is relatively similar across stages 1–3 (~1.3–1.5-fold increase), however there is a higher level in later stage (overall 2-fold increase, Fig. 5B). Most convincingly, individuals who have low MCM10 expression have over a 2-fold increase in overall survival rate (Fig. 5C). Furthermore, individuals with higher levels of MCM10 expression have a 1.6-fold increase in mortality after successful treatment (Fig. 5D). Together, this suggests MCM10 is a clinically relevant prognostic biomarker, with increased expression resulting in poor outcomes.

### MCM10 is crucial for proliferation in an in vitro lung adenocarcinoma model

**Figure 6:**
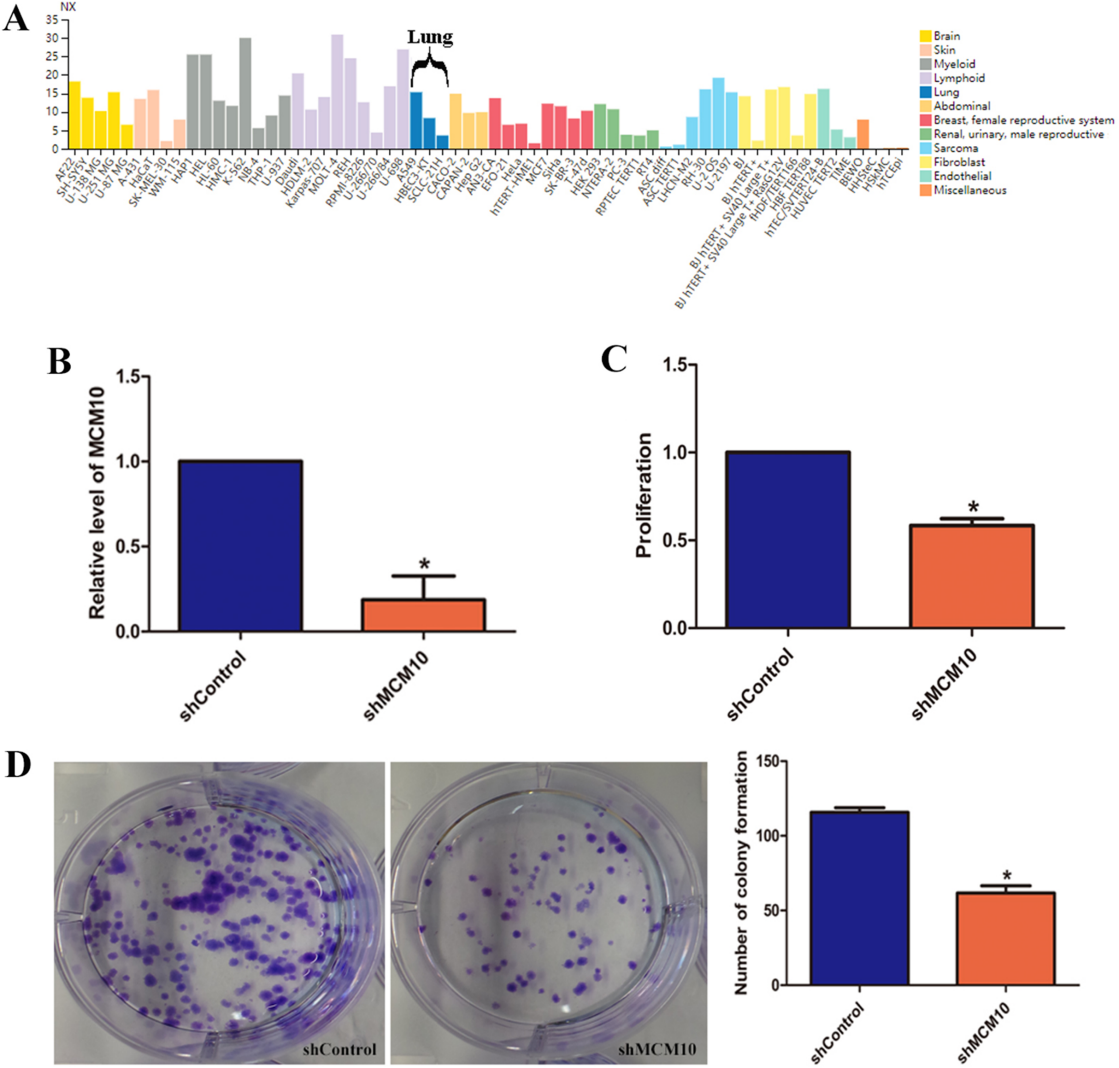
In vitro evaluation of MCM10 in a model of Lung Adenocarcinoma. (A) Several commercially available cell lines have elevated MCM10 levels. A549 cells express MCM10 nearly 50% more than the normal cell line HBEC3-KT. (B) siRNA can be used to effectively decrease MCM10 levels in vitro. Employing this strategy decreases the overall (C) proliferation rates and (D) the self-renewal and colony forming capabilities by 50%.

While these results suggest that MCM10 expression is important in lung adenocarcinoma pathogenesis, they do not fully implicate MCM10 in rampant tumor growth. MCM10 RNA expression is high in a variety of cancer cell lines, with A549 cells demonstrating a much higher level of normalized expression (NX) than the pulmonary epithelial cell line HBEC3 (15.3 vs. 8.3, Fig. 6A). To regulate the MCM10 expression levels, we exposed A549 cell lines to corresponding siRNAs (Fig. 6B). Decreasing the expression resulted in a net loss of 50% of the proliferation capacity (Fig. 6C). Similarly, exposing the A549 cell lines to MCM10 siRNA results in a 50% decrease in colony formation (Fig. 6D). These results demonstrate that MCM10 regulation may be a relevant strategy for therapeutic intervention, and is worth further examination.

### Validation in human samples

**Figure 7:**
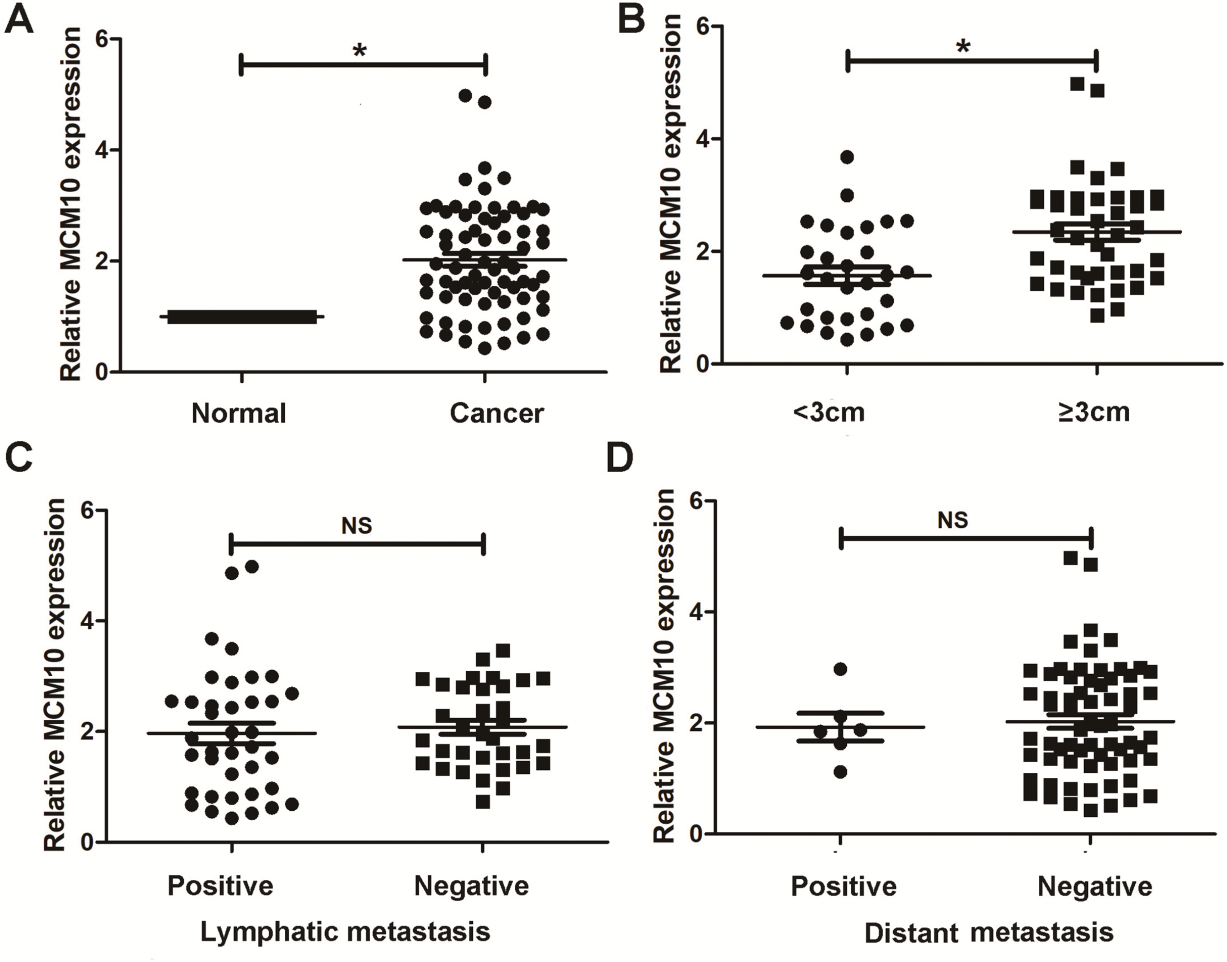
Validation in human samples. MCM10 was validated by 72 pairs clinical biopsy specimens. qPCR was used to determine the correlation between MCM10 expression and tumor presence (A), tumor size (B), lymph metastasis (C) and distant metastasis (D). *P < 0.05.

Thus far, we have determined that MCM10 is a potential diagnostic marker for lung adenocarcinoma and demonstrated upregulation is necessary for excessive tumor growth. We chose to further validate these results using clinical samples taken from patients with lung ACA as well as matching adjacent normal tissue. Results from qPCR align with our bioinformatic results, suggesting lung adenocarcinoma samples have a much higher level of MCM10 than did normal tissue (Fig. 7A). While there was no significant difference due to lymphatic metastasis and distant metastasis (Figs. 7C and 7D), there were significant differences in tumor size (Fig. 7B). These results suggest MCM10 is a valuable marker which can be used to assist in diagnosing lung adenocarcinoma.

## Discussion

Non-small cell lung cancers are the leading cause of cancer related deaths worldwide. Lung adenocarcinoma, which arises from type II alveolar cells, comprises nearly 40% of all lung cancer cases (Zappa & Mousa, 2016). Historically, the prognosis for individuals diagnosed with lung adenocarcinoma has been bleak. However, advances in management strategies have led to a steady decline in overall prevalence. The discovery of prominent diagnostic and prognostic markers have made an impact in the prevalence and overall survival of lung adenocarcinomas cases. While staging of lung adenocarcinoma is crucial for choosing treatment options (Goldstraw et al., 2016), perhaps just as important is identifying tumor subtypes (Saito et al., 2018). Lung adenocarcinoma is notoriously resistant to conventional radio and chemotherapies, presenting a major clinical challenge for clinicians. Personalized strategies based around adjuvant and neo-adjuvant treatments, while controversial, have led to earlier treatment of micrometastases and improved therapeutic compliance, even in advanced stages (Farray, Mirkovic & Albain, 2005). To this end, the goal of the presented work was to discover and evaluate a novel lung adenocarcinoma biomarker.

We used GEO to access three unique genome arrays (Fig. 2). These arrays provided us with data for 367 tumor samples, along with matched data for non-cancer samples. Through an evaluation of the microarray data, we found, while a majority of the expressed genes were only in one data set, 6% (131/2160 DEGs) were found in at least two sets. Furthermore, nearly 2% (41/2160 DEGS) were found in all three data sets. Further evaluation of the 41 co-DEGs using the STRING data base led to the identification of 26 genes whose protein products were highly interconnected. The identification of the HUB genes via the predicted interactions, also known as the interactome (Li, Sahni & Yi, 2016), is a powerful system-level approach that has been used in a variety of contexts to generate hypotheses and shape future research, including lung adenocarcinoma (Selamat et al., 2012), and prostate cancer (Song et al., 2019). Perhaps, not surprisingly, the HUB genes generated from the lung adenocarcinoma samples were highly involved in cellular replication, including mitotic control and DNA replication (Fig. 2D). This is a “hallmark of cancer” and necessary for canonical pathophysiological transformations (Hanahan & Weinberg, 2011).

The degree of connectivity HUB genes can be directly correlated to its involvement in the overall interactome, and especially in cancer, a stronger level of interaction is indicative of involvement in abnormal cell function (Xia et al., 2011). We chose the top 15 most connected HUB genes, as these all had the same predicted degree of connectivity, 26, and evaluated the prevalence of mutation and staging of mutation. Interestingly, while a few of the 15 HUB genes had been evaluated elsewhere for their prognostic value in the context of lung adenocarcinoma (i.e., CDK1 (Shi et al., 2016), BIRC5 (Cho et al., 2015) and FOXM1 (Dai et al., 2015)), several of the HUB genes have only been sparsely covered. We stratified the select HUB genes further by only targeting predicted protein interactions which occur during S-phase of mitosis and DNA replication, as we had previously shown this was a prominent and over represented GO term (Fig. 2D). This resulted in four genes of interest, of which MCM10 was both the most often mutated in the TGCA PanCancer atlas (Fig. 3B) and the most poorly covered in the context of lung adenocarcinoma. The novelty of MCM10 as a potential diagnostic marker made it an enticing target for further study.

MCM10 is a highly conserved protein that is necessary for the formation of the pre-replication complex, formation of replication forks, and recruitment of other proteins required for replication (Baxley & Bielinsky, 2017). MCM10 interacts directly with CDC45, GINS complex proteins and other minichromosome maintenance proteins to form the pre-replication complex (Watase, Takisawa & Kanemaki, 2012). Several of these proteins were identified by STRING as highly connected HUB genes involved in lung adenocarcinoma. MCM10 binds directly to DNA, and suppresses chromosome stability (Thu & Bielinsky, 2014). However, MCM10 is known to be associated with pathological DNA replication, and it has been implicated in multiple cancers, like breast (Mahadevappa et al., 2018) and prostate (Cui et al., 2018).

Several published sources suggest that MCM10 may be mutated in other cancers, however our system-based approach suggested that this may not be the case in lung adenocarcinoma (Figs. 4B and 4C). These results led to the theory that MCM10 dysregulation may be facilitated as a result of cancer progression, but not directly causing the tumorigenesis. Previous work has established MCM10 is regulated by MYCN (Koppen et al., 2007), an oncogene known to be mutated in several subtypes of lung adenocarcinoma (Rickman, Schulte & Eilers, 2018). Therefore, we hypothesize aberrant MYCN function may directly result in amplification of MCM10, which then potentiates lung adenocarcinoma pathogenesis. The association of MCM10 expression and survival demonstrates that it may be a powerful prognostic and diagnostic marker worth further evaluation.

Our analysis of MCM10 in lung adenocarcinoma involved the integration of several bioinformatics approaches. This approach has been applied by other researchers to discover novel HUB genes in a variety of biological processes, including cancer (Fang & Zhang, 2017), developmental biology (Helsen et al., 2019) and vascular disease (Li et al., 2019). However, while bioinformatics approaches provide a convenient and rapid method for processing thousands of samples, it is possible that the results are due to coincidences and not truly linked. Therefore, we further evaluated our results using an in vitro model of lung adenocarcinoma (Fig. 6). These results suggest that MCM10 is necessary for the proliferation of cancer cells. Silencing of MCM10 results in a remarkable decrease in overall proliferation rates, which may be due to both a reduction in pathogenic cell cycle activity and the inability to easily form the pre-replication complex.

## Conclusion

Together, this research demonstrates MCM10 may be both a mediator pathogenic tumor growth and a valuable diagnostic marker. Future work will involve further investigating this marker along with the rest of the pre-replication complex and MYCN, all of which may be involved in NSCLC. We envision this complex being both a critical therapeutic target and a potential fingerprint in identifying lung adenocarcinoma subtypes.

## Supplemental Information

10.7717/peerj.10560/supp-1 Supplemental Information 1 CCK8.

10.7717/peerj.10560/supp-2 Supplemental Information 2 Colony formation.

10.7717/peerj.10560/supp-3 Supplemental Information 3 KEGG.

10.7717/peerj.10560/supp-4 Supplemental Information 4 PCR.

10.7717/peerj.10560/supp-5 Supplemental Information 5 String interactions default node.

10.7717/peerj.10560/supp-6 Supplemental Information 6 Biochip information.

## Additional Information and Declarations

### Data Availability

The following information was supplied regarding data availability: The raw measurements are available in the Supplemental Files.
